# Supplementary material for: Investigating the causal interplay between sleep traits and risk of acute myocardial infarction: a Mendelian randomization study
Source: BMC Med. 2023 Oct 5;21:385. doi: 10.1186/s12916-023-03078-0 (PMC10557341; doi:10.1186/s12916-023-03078-0)
Supplement: Supplementary file 2 — Additional file 2. Genetic variants. Table G1. Summary information of genetic variants identified for insomnia symptoms. Table G2. Summary information of genetic variants identified for sleep duration. Table G3. Summary information of genetic variants identified for short sleep. Table G4. Summary information of genetic variants identified for long sleep. Table G5. Summary information of genetic variants identified for chronotype. [file 12916_2023_3078_MOESM2_ESM.docx]

**Genetic variants**

Table G1: Summary information of genetic variants identified for insomnia symptoms.

| Sr. No. | rsID | Effect Allele | Other Allele | Beta | Standard Error | P | Effect Allele Frequency | N | Replication in 23andMe |
| --- | --- | --- | --- | --- | --- | --- | --- | --- | --- |
|  | rs113851554 | T | G | 0.2062 | 0.014 | 1.56E-51 | 0.0506 | 1 331 010 | Yes |
|  | rs62149809 | A | G | 0.1476 | 0.025 | 5.71E-09 | 0.986 | 1 331 010 | No |
|  | rs1064939 | A | T | 0.1302 | 0.02 | 2.16E-10 | 0.9784 | 1 331 010 | No |
|  | rs79204944 | A | G | 0.0788 | 0.014 | 4.24E-08 | 0.0449 | 1 331 010 | No |
|  | rs72899452 | T | C | 0.0742 | 0.012 | 1.00E-09 | 0.0648 | 1 331 010 | Yes |
|  | rs55972276 | A | C | 0.0733 | 0.009 | 4.19E-17 | 0.1366 | 1 331 010 | Yes |
|  | rs77641763 | T | C | 0.0714 | 0.009 | 6.53E-15 | 0.122 | 1 331 010 | Yes |
|  | rs138014720 | A | T | 0.0695 | 0.013 | 3.46E-08 | 0.9406 | 1 331 010 | No |
|  | rs2286729 | A | G | 0.0695 | 0.011 | 5.37E-11 | 0.0862 | 1 331 010 | Yes |
|  | rs118166957 | T | C | 0.0677 | 0.008 | 1.95E-16 | 0.1591 | 1 331 010 | Yes |
|  | rs11650304 | C | G | 0.0667 | 0.012 | 1.23E-08 | 0.9308 | 1 331 010 | No |
|  | rs62158170 | A | G | 0.0658 | 0.007 | 1.20E-19 | 0.7856 | 1 331 010 | Yes |
|  | rs62264767 | A | C | 0.0649 | 0.008 | 1.63E-14 | 0.8531 | 1 331 010 | Yes |
|  | rs7168238 | C | G | 0.0639 | 0.011 | 1.80E-08 | 0.0743 | 1 331 010 | No |
|  | rs699844 | A | G | 0.0602 | 0.011 | 4.11E-08 | 0.9195 | 1 331 010 | Yes |
|  | rs7566062 | T | C | 0.0592 | 0.007 | 1.37E-16 | 0.2248 | 1 331 010 | Yes |
|  | rs28611339 | T | G | 0.0583 | 0.009 | 8.46E-11 | 0.1282 | 1 331 010 | No |
|  | rs1015438 | A | G | 0.0583 | 0.008 | 2.51E-14 | 0.1882 | 1 331 010 | Yes |
|  | rs6465151 | T | C | 0.0564 | 0.009 | 1.90E-09 | 0.1134 | 1 331 010 | No |
|  | rs2815757 | T | C | 0.0554 | 0.008 | 2.24E-13 | 0.8089 | 1 331 010 | Yes |
|  | rs16903122 | T | C | 0.0554 | 0.007 | 9.04E-16 | 0.2487 | 1 331 010 | Yes |
|  | rs2792990 | C | G | 0.0545 | 0.008 | 1.15E-10 | 0.8553 | 1 331 010 | Yes |
|  | rs62590551 | A | G | 0.0545 | 0.01 | 4.61E-09 | 0.9029 | 1 331 010 | Yes |
|  | rs34490907 | C | G | 0.0535 | 0.009 | 1.76E-08 | 0.8878 | 1 331 010 | No |
|  | rs670501 | T | C | 0.0526 | 0.007 | 7.40E-13 | 0.2133 | 1 331 010 | Yes |
|  | rs1927902 | T | C | 0.0526 | 0.007 | 1.15E-14 | 0.2542 | 1 331 010 | Yes |
|  | rs1620977 | A | G | 0.0516 | 0.007 | 2.27E-14 | 0.2696 | 1 331 010 | Yes |
|  | rs75452188 | A | G | 0.0516 | 0.009 | 1.58E-08 | 0.878 | 1 331 010 | No |
|  | rs71575448 | A | G | 0.0507 | 0.009 | 3.38E-09 | 0.8602 | 1 331 010 | No |
|  | rs11756035 | C | G | 0.0507 | 0.009 | 1.29E-08 | 0.1281 | 1 331 010 | No |
|  | rs62429521 | A | C | 0.0507 | 0.008 | 1.78E-09 | 0.1456 | 1 331 010 | No |
|  | rs7992992 | A | G | 0.0507 | 0.009 | 1.15E-08 | 0.1286 | 1 331 010 | Yes |
|  | rs908668 | T | C | 0.0497 | 0.007 | 1.41E-11 | 0.2081 | 1 331 010 | Yes |
|  | rs2491124 | T | C | 0.0488 | 0.006 | 8.81E-16 | 0.5758 | 1 331 010 | Yes |
|  | rs35322724 | A | C | 0.0488 | 0.006 | 3.75E-16 | 0.5774 | 1 331 010 | Yes |
|  | rs62068188 | T | C | 0.0488 | 0.008 | 1.18E-09 | 0.8339 | 1 331 010 | No |
|  | rs9931543 | T | C | 0.0478 | 0.007 | 1.11E-12 | 0.7359 | 1 331 010 | Yes |
|  | rs3902952 | T | C | 0.0478 | 0.008 | 2.55E-10 | 0.1881 | 1 331 010 | Yes |
|  | rs4790076 | T | C | 0.0478 | 0.008 | 1.76E-09 | 0.1742 | 1 331 010 | No |
|  | rs45453598 | A | T | 0.0469 | 0.008 | 4.42E-09 | 0.169 | 1 331 010 | Yes |
|  | rs17223714 | A | G | 0.0459 | 0.007 | 2.44E-10 | 0.7885 | 1 331 010 | Yes |
|  | rs9316619 | T | C | 0.0459 | 0.008 | 5.50E-09 | 0.8249 | 1 331 010 | No |
|  | rs429358 | T | C | 0.0459 | 0.008 | 2.13E-08 | 0.8458 | 1 331 010 | No |
|  | rs12310246 | A | G | 0.0450 | 0.007 | 4.74E-11 | 0.2489 | 1 331 010 | No |
|  | rs830716 | C | G | 0.0450 | 0.007 | 8.68E-12 | 0.7133 | 1 331 010 | Yes |
|  | rs67501351 | C | G | 0.0450 | 0.007 | 5.36E-11 | 0.7451 | 1 331 010 | No |
|  | rs34214423 | A | C | 0.0450 | 0.008 | 3.18E-09 | 0.8087 | 1 331 010 | No |
|  | rs12614369 | A | G | 0.0440 | 0.008 | 7.21E-09 | 0.8158 | 1 331 010 | No |
|  | rs116466468 | T | C | 0.0440 | 0.007 | 2.11E-10 | 0.7593 | 1 331 010 | No |
|  | rs2903385 | A | G | 0.0431 | 0.006 | 4.53E-13 | 0.4844 | 1 331 010 | Yes |
|  | rs6606731 | A | T | 0.0431 | 0.008 | 1.51E-08 | 0.1923 | 1 331 010 | No |
|  | rs742760 | A | T | 0.0431 | 0.008 | 2.48E-08 | 0.8155 | 1 331 010 | Yes |
|  | rs10800992 | T | C | 0.0421 | 0.006 | 3.84E-12 | 0.4431 | 1 331 010 | Yes |
|  | rs55772859 | A | C | 0.0421 | 0.006 | 4.82E-11 | 0.3106 | 1 331 010 | Yes |
|  | rs10865954 | T | C | 0.0421 | 0.006 | 1.92E-11 | 0.3344 | 1 331 010 | No |
|  | rs17005118 | A | G | 0.0421 | 0.007 | 6.13E-10 | 0.264 | 1 331 010 | No |
|  | rs35539975 | A | G | 0.0421 | 0.007 | 4.49E-09 | 0.7785 | 1 331 010 | No |
|  | rs12666306 | A | G | 0.0421 | 0.006 | 2.24E-12 | 0.5017 | 1 331 010 | Yes |
|  | rs2867690 | T | C | 0.0421 | 0.008 | 3.70E-08 | 0.1819 | 1 331 010 | No |
|  | rs12030482 | A | T | 0.0411 | 0.007 | 8.16E-09 | 0.2201 | 1 331 010 | No |
|  | rs17025198 | A | G | 0.0411 | 0.007 | 2.19E-08 | 0.2043 | 1 331 010 | No |
|  | rs56133505 | A | G | 0.0411 | 0.006 | 5.59E-12 | 0.5369 | 1 331 010 | Yes |
|  | rs7486418 | T | G | 0.0411 | 0.006 | 6.84E-11 | 0.657 | 1 331 010 | No |
|  | rs715338 | A | G | 0.0411 | 0.006 | 7.85E-12 | 0.5776 | 1 331 010 | Yes |
|  | rs4643373 | T | C | 0.0411 | 0.007 | 1.58E-10 | 0.7006 | 1 331 010 | No |
|  | rs56097173 | T | C | 0.0402 | 0.006 | 2.69E-10 | 0.6808 | 1 331 010 | No |
|  | rs12991815 | C | G | 0.0402 | 0.006 | 3.02E-11 | 0.4241 | 1 331 010 | Yes |
|  | rs12187443 | T | C | 0.0402 | 0.006 | 1.64E-10 | 0.6681 | 1 331 010 | No |
|  | rs9373590 | A | T | 0.0402 | 0.006 | 2.18E-11 | 0.5078 | 1 331 010 | No |
|  | rs4592425 | T | G | 0.0402 | 0.006 | 4.31E-10 | 0.6965 | 1 331 010 | No |
|  | rs11149313 | A | G | 0.0402 | 0.007 | 2.38E-09 | 0.7297 | 1 331 010 | No |
|  | rs6019663 | T | C | 0.0402 | 0.007 | 6.47E-10 | 0.293 | 1 331 010 | No |
|  | rs62194948 | C | G | 0.0392 | 0.007 | 4.64E-09 | 0.275 | 1 331 010 | Yes |
|  | rs6808140 | T | C | 0.0392 | 0.006 | 5.35E-11 | 0.5053 | 1 331 010 | Yes |
|  | rs35110063 | A | G | 0.0392 | 0.006 | 8.82E-11 | 0.4266 | 1 331 010 | Yes |
|  | rs1147852 | A | G | 0.0392 | 0.006 | 9.94E-10 | 0.3095 | 1 331 010 | Yes |
|  | rs324017 | A | C | 0.0392 | 0.007 | 1.61E-09 | 0.294 | 1 331 010 | No |
|  | rs6562066 | T | C | 0.0392 | 0.006 | 1.38E-10 | 0.3688 | 1 331 010 | No |
|  | rs1038093 | T | C | 0.0392 | 0.006 | 2.47E-10 | 0.6282 | 1 331 010 | Yes |
|  | rs11090039 | A | G | 0.0392 | 0.007 | 1.82E-09 | 0.2871 | 1 331 010 | No |
|  | rs1861412 | A | G | 0.0383 | 0.006 | 1.67E-10 | 0.4338 | 1 331 010 | No |
|  | rs6888135 | A | C | 0.0383 | 0.006 | 1.21E-10 | 0.4965 | 1 331 010 | No |
|  | rs940780 | T | C | 0.0383 | 0.006 | 8.50E-10 | 0.3588 | 1 331 010 | No |
|  | rs12924275 | T | C | 0.0383 | 0.007 | 1.93E-08 | 0.268 | 1 331 010 | No |
|  | rs2398144 | A | C | 0.0383 | 0.006 | 5.09E-10 | 0.3947 | 1 331 010 | Yes |
|  | rs11679943 | A | G | 0.0373 | 0.006 | 3.16E-09 | 0.3471 | 1 331 010 | Yes |
|  | rs6756610 | C | G | 0.0373 | 0.006 | 1.14E-09 | 0.6291 | 1 331 010 | No |
|  | rs62213452 | T | G | 0.0373 | 0.007 | 2.39E-08 | 0.2788 | 1 331 010 | No |
|  | rs7040224 | A | G | 0.0373 | 0.006 | 4.24E-09 | 0.3161 | 1 331 010 | No |
|  | rs72773790 | T | C | 0.0373 | 0.006 | 3.71E-09 | 0.6732 | 1 331 010 | No |
|  | rs5877 | T | C | 0.0363 | 0.006 | 1.23E-08 | 0.6691 | 1 331 010 | No |
|  | rs1530938 | A | G | 0.0363 | 0.006 | 8.82E-10 | 0.4423 | 1 331 010 | No |
|  | rs7625896 | A | G | 0.0363 | 0.006 | 5.28E-09 | 0.6545 | 1 331 010 | No |
|  | rs1264419 | C | G | 0.0363 | 0.006 | 8.91E-10 | 0.5128 | 1 331 010 | Yes |
|  | rs2737240 | A | G | 0.0363 | 0.007 | 3.37E-08 | 0.7076 | 1 331 010 | No |
|  | rs10756571 | T | C | 0.0363 | 0.006 | 1.80E-08 | 0.6853 | 1 331 010 | No |
|  | rs2221119 | C | G | 0.0363 | 0.006 | 2.00E-09 | 0.443 | 1 331 010 | No |
|  | rs9540729 | A | T | 0.0363 | 0.006 | 1.40E-09 | 0.4794 | 1 331 010 | Yes |
|  | rs34967082 | A | G | 0.0354 | 0.006 | 4.34E-09 | 0.4136 | 1 331 010 | No |
|  | rs2216427 | C | G | 0.0354 | 0.006 | 1.60E-08 | 0.6527 | 1 331 010 | Yes |
|  | rs6601080 | A | G | 0.0354 | 0.006 | 2.21E-08 | 0.6762 | 1 331 010 | No |
|  | rs2598293 | T | C | 0.0354 | 0.006 | 2.48E-09 | 0.4763 | 1 331 010 | No |
|  | rs871994 | A | C | 0.0354 | 0.006 | 5.50E-09 | 0.4352 | 1 331 010 | Yes |
|  | rs1167132 | T | C | 0.0354 | 0.006 | 8.73E-09 | 0.3917 | 1 331 010 | Yes |
|  | rs176644 | T | G | 0.0354 | 0.006 | 9.49E-09 | 0.4036 | 1 331 010 | No |
|  | rs12605642 | T | G | 0.0354 | 0.006 | 2.13E-09 | 0.4864 | 1 331 010 | No |
|  | rs9964420 | A | C | 0.0354 | 0.007 | 4.54E-08 | 0.301 | 1 331 010 | Yes |
|  | rs72820274 | A | G | 0.0344 | 0.006 | 1.28E-08 | 0.417 | 1 331 010 | No |
|  | rs10928256 | T | C | 0.0344 | 0.006 | 1.61E-08 | 0.4192 | 1 331 010 | No |
|  | rs4260410 | T | C | 0.0344 | 0.006 | 4.87E-08 | 0.332 | 1 331 010 | Yes |
|  | rs11722569 | T | C | 0.0344 | 0.006 | 2.91E-08 | 0.6586 | 1 331 010 | Yes |
|  | rs13138995 | A | G | 0.0344 | 0.006 | 1.97E-08 | 0.3896 | 1 331 010 | No |
|  | rs6978112 | T | C | 0.0344 | 0.006 | 2.11E-08 | 0.4105 | 1 331 010 | No |
|  | rs2030672 | C | G | 0.0344 | 0.006 | 1.10E-08 | 0.5589 | 1 331 010 | No |
|  | rs874168 | T | C | 0.0344 | 0.006 | 7.95E-09 | 0.5254 | 1 331 010 | Yes |
|  | rs10898940 | A | C | 0.0344 | 0.006 | 8.09E-09 | 0.5173 | 1 331 010 | No |
|  | rs1567084 | A | G | 0.0334 | 0.006 | 2.14E-08 | 0.4981 | 1 331 010 | No |
|  | rs1580173 | A | G | 0.0334 | 0.006 | 2.28E-08 | 0.5608 | 1 331 010 | No |
|  | rs1357685 | T | C | 0.0334 | 0.006 | 1.39E-08 | 0.4735 | 1 331 010 | No |
|  | rs4588900 | A | G | 0.0334 | 0.006 | 1.57E-08 | 0.5164 | 1 331 010 | No |
|  | rs28552587 | A | G | 0.0334 | 0.006 | 3.30E-08 | 0.5641 | 1 331 010 | No |
|  | rs10955647 | T | G | 0.0334 | 0.006 | 1.84E-08 | 0.5321 | 1 331 010 | No |
|  | rs6597649 | T | C | 0.0334 | 0.006 | 3.05E-08 | 0.3994 | 1 331 010 | No |
|  | rs10825503 | T | G | 0.0334 | 0.006 | 1.43E-08 | 0.4873 | 1 331 010 | No |
|  | rs667730 | T | C | 0.0334 | 0.006 | 2.26E-08 | 0.5788 | 1 331 010 | Yes |
|  | rs647905 | T | C | 0.0334 | 0.006 | 2.87E-08 | 0.5409 | 1 331 010 | Yes |
|  | rs10947987 | T | C | -0.0325 | 0.006 | 4.08E-08 | 0.4431 | 1 331 010 | No |
|  | rs4858708 | A | T | -0.0336 | 0.006 | 1.23E-08 | 0.5305 | 1 331 010 | No |
|  | rs2364921 | T | C | -0.0336 | 0.006 | 2.13E-08 | 0.4691 | 1 331 010 | No |
|  | rs238869 | T | C | -0.0336 | 0.006 | 3.36E-08 | 0.6229 | 1 331 010 | No |
|  | rs190073 | A | G | -0.0336 | 0.006 | 2.86E-08 | 0.4141 | 1 331 010 | No |
|  | rs9563886 | T | C | -0.0336 | 0.006 | 3.08E-08 | 0.6063 | 1 331 010 | No |
|  | rs2447094 | A | C | -0.0336 | 0.006 | 2.50E-08 | 0.4687 | 1 331 010 | No |
|  | rs1553754 | T | G | -0.0336 | 0.006 | 3.51E-08 | 0.5621 | 1 331 010 | Yes |
|  | rs11588755 | A | G | -0.0346 | 0.006 | 5.14E-09 | 0.522 | 1 331 010 | No |
|  | rs11119409 | T | C | -0.0346 | 0.006 | 1.19E-08 | 0.5866 | 1 331 010 | No |
|  | rs728017 | A | G | -0.0346 | 0.006 | 9.51E-09 | 0.3864 | 1 331 010 | No |
|  | rs1731951 | A | T | -0.0346 | 0.006 | 1.36E-08 | 0.4434 | 1 331 010 | No |
|  | rs4788203 | A | G | -0.0346 | 0.006 | 6.32E-09 | 0.4334 | 1 331 010 | Yes |
|  | rs12454003 | C | G | -0.0346 | 0.006 | 4.94E-09 | 0.4821 | 1 331 010 | No |
|  | rs910187 | A | G | -0.0346 | 0.006 | 1.63E-08 | 0.373 | 1 331 010 | No |
|  | rs34036083 | T | C | -0.0356 | 0.006 | 2.07E-08 | 0.6576 | 1 331 010 | No |
|  | rs12520974 | T | C | -0.0356 | 0.006 | 1.69E-09 | 0.4846 | 1 331 010 | No |
|  | rs701394 | A | G | -0.0356 | 0.006 | 6.83E-09 | 0.6376 | 1 331 010 | No |
|  | rs37445 | A | G | -0.0356 | 0.006 | 4.88E-09 | 0.3905 | 1 331 010 | No |
|  | rs17367725 | T | C | -0.0356 | 0.006 | 9.29E-09 | 0.3513 | 1 331 010 | Yes |
|  | rs9469434 | C | G | -0.0356 | 0.007 | 4.41E-08 | 0.2851 | 1 331 010 | Yes |
|  | rs10758593 | A | G | -0.0356 | 0.006 | 4.90E-09 | 0.3989 | 1 331 010 | Yes |
|  | rs7402939 | T | C | -0.0356 | 0.006 | 5.19E-09 | 0.376 | 1 331 010 | Yes |
|  | rs2838787 | A | G | -0.0356 | 0.006 | 7.65E-09 | 0.3924 | 1 331 010 | No |
|  | rs6702604 | A | G | -0.0367 | 0.006 | 1.30E-09 | 0.5843 | 1 331 010 | No |
|  | rs823247 | T | C | -0.0367 | 0.006 | 5.25E-10 | 0.479 | 1 331 010 | No |
|  | rs1519102 | C | G | -0.0367 | 0.006 | 1.90E-08 | 0.6891 | 1 331 010 | No |
|  | rs1064213 | A | G | -0.0367 | 0.006 | 6.41E-10 | 0.4789 | 1 331 010 | Yes |
|  | rs7599697 | T | C | -0.0367 | 0.006 | 5.00E-09 | 0.3583 | 1 331 010 | Yes |
|  | rs2388840 | A | G | -0.0367 | 0.006 | 1.37E-09 | 0.5757 | 1 331 010 | Yes |
|  | rs7475916 | C | G | -0.0367 | 0.006 | 6.70E-09 | 0.3533 | 1 331 010 | No |
|  | rs4767645 | T | G | -0.0367 | 0.006 | 6.47E-10 | 0.4614 | 1 331 010 | No |
|  | rs6510033 | A | G | -0.0367 | 0.007 | 4.66E-08 | 0.7253 | 1 331 010 | Yes |
|  | rs623025 | T | C | -0.0377 | 0.007 | 3.16E-08 | 0.2552 | 1 331 010 | No |
|  | rs73163783 | T | C | -0.0377 | 0.007 | 1.39E-08 | 0.7232 | 1 331 010 | No |
|  | rs10944696 | A | G | -0.0377 | 0.007 | 7.99E-09 | 0.2978 | 1 331 010 | No |
|  | rs6973090 | A | G | -0.0377 | 0.007 | 4.31E-08 | 0.25 | 1 331 010 | No |
|  | rs671985 | A | G | -0.0377 | 0.006 | 2.79E-10 | 0.4516 | 1 331 010 | No |
|  | rs11001276 | A | T | -0.0377 | 0.007 | 2.52E-08 | 0.74 | 1 331 010 | No |
|  | rs214934 | A | T | -0.0377 | 0.006 | 3.16E-09 | 0.3123 | 1 331 010 | Yes |
|  | rs6589988 | A | G | -0.0377 | 0.006 | 4.70E-09 | 0.6757 | 1 331 010 | Yes |
|  | rs8181889 | A | G | -0.0377 | 0.006 | 8.90E-10 | 0.3992 | 1 331 010 | No |
|  | rs1536053 | T | C | -0.0377 | 0.006 | 6.04E-09 | 0.3157 | 1 331 010 | Yes |
|  | rs3184470 | A | G | -0.0377 | 0.006 | 9.73E-10 | 0.3507 | 1 331 010 | No |
|  | rs8076183 | T | C | -0.0377 | 0.006 | 2.75E-10 | 0.4485 | 1 331 010 | Yes |
|  | rs1937447 | C | G | -0.0387 | 0.007 | 2.08E-08 | 0.7593 | 1 331 010 | Yes |
|  | rs7571486 | A | G | -0.0387 | 0.007 | 1.40E-08 | 0.251 | 1 331 010 | No |
|  | rs4502882 | T | C | -0.0387 | 0.006 | 7.96E-10 | 0.6578 | 1 331 010 | Yes |
|  | rs6457796 | T | C | -0.0387 | 0.007 | 1.12E-08 | 0.7313 | 1 331 010 | No |
|  | rs4090240 | T | C | -0.0387 | 0.007 | 8.46E-09 | 0.2775 | 1 331 010 | Yes |
|  | rs12251016 | A | T | -0.0387 | 0.006 | 3.89E-10 | 0.6559 | 1 331 010 | Yes |
|  | rs224029 | T | C | -0.0387 | 0.006 | 2.51E-10 | 0.3995 | 1 331 010 | No |
|  | rs566673 | T | G | -0.0387 | 0.006 | 1.18E-10 | 0.5351 | 1 331 010 | Yes |
|  | rs10502966 | A | G | -0.0387 | 0.006 | 8.54E-11 | 0.582 | 1 331 010 | Yes |
|  | rs7615602 | C | G | -0.0398 | 0.007 | 2.59E-09 | 0.2715 | 1 331 010 | No |
|  | rs521484 | A | G | -0.0398 | 0.007 | 1.53E-08 | 0.7668 | 1 331 010 | Yes |
|  | rs75932578 | T | C | -0.0398 | 0.007 | 4.15E-08 | 0.2159 | 1 331 010 | No |
|  | rs12790660 | T | C | -0.0398 | 0.006 | 4.49E-10 | 0.6844 | 1 331 010 | No |
|  | rs2389631 | A | C | -0.0398 | 0.006 | 2.03E-10 | 0.6666 | 1 331 010 | Yes |
|  | rs2089358 | T | C | -0.0408 | 0.007 | 2.75E-10 | 0.7038 | 1 331 010 | Yes |
|  | rs1289939 | T | C | -0.0408 | 0.007 | 6.00E-09 | 0.2325 | 1 331 010 | No |
|  | rs11803128 | A | G | -0.0408 | 0.006 | 6.85E-11 | 0.6541 | 1 331 010 | Yes |
|  | rs6545798 | A | T | -0.0408 | 0.006 | 1.19E-11 | 0.4104 | 1 331 010 | Yes |
|  | rs4664299 | T | C | -0.0408 | 0.007 | 4.95E-09 | 0.2349 | 1 331 010 | Yes |
|  | rs3774751 | T | G | -0.0408 | 0.006 | 7.32E-12 | 0.4621 | 1 331 010 | Yes |
|  | rs7044885 | C | G | -0.0408 | 0.006 | 5.67E-12 | 0.4417 | 1 331 010 | Yes |
|  | rs6734957 | T | G | -0.0419 | 0.007 | 1.82E-09 | 0.2388 | 1 331 010 | Yes |
|  | rs62301574 | C | G | -0.0419 | 0.007 | 1.37E-08 | 0.7996 | 1 331 010 | Yes |
|  | rs12917449 | A | C | -0.0419 | 0.008 | 2.97E-08 | 0.8061 | 1 331 010 | No |
|  | rs9889282 | A | C | -0.0419 | 0.006 | 4.70E-12 | 0.6129 | 1 331 010 | Yes |
|  | rs11126082 | C | G | -0.0429 | 0.006 | 8.26E-13 | 0.4398 | 1 331 010 | Yes |
|  | rs984306 | T | C | -0.0429 | 0.007 | 7.94E-10 | 0.7547 | 1 331 010 | No |
|  | rs314281 | T | C | -0.0429 | 0.006 | 6.03E-13 | 0.4531 | 1 331 010 | No |
|  | rs10761240 | A | G | -0.0429 | 0.006 | 2.12E-12 | 0.3963 | 1 331 010 | Yes |
|  | rs12912299 | T | C | -0.0429 | 0.006 | 4.42E-13 | 0.4893 | 1 331 010 | Yes |
|  | rs4238755 | A | C | -0.0429 | 0.007 | 2.30E-10 | 0.2638 | 1 331 010 | Yes |
|  | rs60565673 | T | G | -0.0429 | 0.006 | 1.59E-12 | 0.6211 | 1 331 010 | Yes |
|  | rs12983032 | A | G | -0.0429 | 0.006 | 1.07E-11 | 0.3434 | 1 331 010 | No |
|  | rs694786 | T | C | -0.0440 | 0.006 | 1.97E-13 | 0.4605 | 1 331 010 | Yes |
|  | rs17083297 | A | C | -0.0440 | 0.008 | 1.60E-08 | 0.1766 | 1 331 010 | No |
|  | rs3131638 | A | G | -0.0440 | 0.007 | 7.88E-10 | 0.2261 | 1 331 010 | No |
|  | rs6967168 | T | G | -0.0440 | 0.007 | 1.39E-10 | 0.7544 | 1 331 010 | No |
|  | rs524859 | A | G | -0.0440 | 0.006 | 1.48E-12 | 0.3601 | 1 331 010 | No |
|  | rs61921611 | T | C | -0.0440 | 0.006 | 7.84E-12 | 0.692 | 1 331 010 | Yes |
|  | rs7214267 | A | G | -0.0440 | 0.006 | 5.09E-13 | 0.581 | 1 331 010 | No |
|  | rs61765555 | T | C | -0.0450 | 0.007 | 4.00E-11 | 0.2552 | 1 331 010 | Yes |
|  | rs11605348 | A | G | -0.0450 | 0.006 | 7.01E-13 | 0.3495 | 1 331 010 | Yes |
|  | rs16990210 | T | C | -0.0460 | 0.008 | 1.97E-08 | 0.8478 | 1 331 010 | No |
|  | rs12540241 | A | T | -0.0460 | 0.008 | 1.58E-09 | 0.1926 | 1 331 010 | No |
|  | rs10947690 | A | G | -0.0471 | 0.007 | 4.04E-12 | 0.7408 | 1 331 010 | Yes |
|  | rs4702 | A | G | -0.0481 | 0.006 | 6.78E-16 | 0.5562 | 1 331 010 | Yes |
|  | rs73079014 | T | C | -0.0492 | 0.009 | 3.65E-08 | 0.126 | 1 331 010 | No |
|  | rs8180817 | C | G | -0.0492 | 0.006 | 1.83E-16 | 0.4304 | 1 331 010 | Yes |
|  | rs76145129 | T | G | -0.0502 | 0.009 | 2.73E-08 | 0.1238 | 1 331 010 | No |
|  | rs1031654 | A | C | -0.0513 | 0.007 | 3.88E-12 | 0.7996 | 1 331 010 | No |
|  | rs152555 | A | G | -0.0523 | 0.008 | 4.83E-10 | 0.8544 | 1 331 010 | No |
|  | rs2431108 | T | C | -0.0534 | 0.006 | 7.83E-17 | 0.672 | 1 331 010 | Yes |
|  | rs9394502 | T | C | -0.0545 | 0.006 | 7.76E-18 | 0.3343 | 1 331 010 | Yes |
|  | rs4709655 | T | C | -0.0545 | 0.009 | 3.09E-09 | 0.1191 | 1 331 010 | Yes |
|  | rs28582096 | A | G | -0.0545 | 0.007 | 1.74E-13 | 0.205 | 1 331 010 | Yes |
|  | rs4981170 | A | G | -0.0545 | 0.008 | 7.33E-13 | 0.1943 | 1 331 010 | Yes |
|  | rs72657797 | T | C | -0.0555 | 0.008 | 1.52E-12 | 0.1759 | 1 331 010 | Yes |
|  | rs8180457 | T | C | -0.0555 | 0.008 | 1.12E-11 | 0.1573 | 1 331 010 | Yes |
|  | rs73671843 | A | G | -0.0555 | 0.009 | 5.49E-10 | 0.1257 | 1 331 010 | No |
|  | rs17324524 | T | C | -0.0576 | 0.009 | 5.01E-10 | 0.883 | 1 331 010 | Yes |
|  | rs13010288 | T | G | -0.0598 | 0.009 | 9.26E-12 | 0.1326 | 1 331 010 | Yes |
|  | rs62383308 | A | G | -0.0598 | 0.011 | 3.98E-08 | 0.0805 | 1 331 010 | Yes |
|  | rs17643634 | T | C | -0.0598 | 0.008 | 1.34E-13 | 0.165 | 1 331 010 | Yes |
|  | rs66674044 | A | T | -0.0598 | 0.009 | 2.18E-12 | 0.8573 | 1 331 010 | Yes |
|  | rs6119267 | C | G | -0.0598 | 0.006 | 2.32E-20 | 0.6891 | 1 331 010 | Yes |
|  | rs492858 | T | C | -0.0661 | 0.011 | 3.46E-09 | 0.0759 | 1 331 010 | Yes |
|  | rs10947428 | T | C | -0.0683 | 0.007 | 9.06E-21 | 0.7858 | 1 331 010 | Yes |
|  | rs79693059 | C | G | -0.0726 | 0.011 | 1.61E-11 | 0.9158 | 1 331 010 | Yes |
|  | rs9527083 | A | G | -0.0758 | 0.006 | 1.61E-32 | 0.6705 | 1 331 010 | Yes |
|  | rs11838830 | A | G | -0.0801 | 0.013 | 5.20E-10 | 0.9436 | 1 331 010 | Yes |
|  | rs4699157 | T | C | -0.0812 | 0.015 | 3.98E-08 | 0.958 | 1 331 010 | No |
|  | rs7432782 | T | C | -0.0834 | 0.014 | 7.42E-09 | 0.9558 | 1 331 010 | No |
|  | rs13135092 | A | G | -0.0888 | 0.011 | 2.53E-16 | 0.9175 | 1 331 010 | Yes |
|  | rs17520265 | A | G | -0.0910 | 0.016 | 2.87E-08 | 0.0342 | 1 331 010 | No |
|  | rs78206187 | A | G | -0.0943 | 0.013 | 2.96E-13 | 0.9442 | 1 331 010 | No |
|  | rs117630493 | C | G | -0.1009 | 0.018 | 3.61E-08 | 0.9726 | 1 331 010 | Yes |
|  | rs138678612 | A | G | -0.1165 | 0.02 | 1.41E-08 | 0.9783 | 1 331 010 | No |

Table G2: Summary information of genetic variants identified for sleep duration.

| Sr. No. | rsID | Effect Allele | Other Allele | Beta | Standard Error | P | Effect Allele Frequency | N |
| --- | --- | --- | --- | --- | --- | --- | --- | --- |
|  | rs7556815 | A | G | 2.443 | 0.164 | 1.3E-49 | 0.219 | 446 118 |
|  | rs75539574 | C | A | 2.175 | 0.244 | 6.9E-19 | 0.086 | 446 118 |
|  | rs12607679 | T | C | 1.208 | 0.156 | 8.3E-15 | 0.738 | 446 118 |
|  | rs915416 | C | G | 1.156 | 0.150 | 9.9E-15 | 0.290 | 446 118 |
|  | rs9940646 | C | G | 1.017 | 0.137 | 1.2E-13 | 0.578 | 446 118 |
|  | rs13109404 | T | G | 1.872 | 0.264 | 1.4E-12 | 0.928 | 446 118 |
|  | rs8050478 | G | A | 0.960 | 0.136 | 1.7E-12 | 0.500 | 446 118 |
|  | rs56372231 | T | C | 1.017 | 0.144 | 2.2E-12 | 0.334 | 446 118 |
|  | rs13088093 | G | T | 0.976 | 0.144 | 7E-12 | 0.336 | 446 118 |
|  | rs2079070 | C | G | 1.053 | 0.154 | 7.5E-12 | 0.265 | 446 118 |
|  | rs34556183 | A | G | 1.015 | 0.151 | 2.3E-11 | 0.720 | 446 118 |
|  | rs3095508 | C | A | 0.921 | 0.138 | 3.1E-11 | 0.594 | 446 118 |
|  | rs34731055 | T | C | 1.168 | 0.177 | 3.7E-11 | 0.181 | 446 118 |
|  | rs73219758 | G | A | 0.984 | 0.150 | 5.6E-11 | 0.708 | 446 118 |
|  | rs10973207 | T | G | 1.226 | 0.187 | 6E-11 | 0.158 | 446 118 |
|  | rs2139261 | G | C | 1.122 | 0.174 | 8.50E-11 | 0.749 | 446 118 |
|  | rs4592416 | G | A | 0.881 | 0.136 | 9.3E-11 | 0.464 | 446 118 |
|  | rs365663 | A | G | 0.878 | 0.137 | 1E-10 | 0.546 | 446 118 |
|  | rs1517572 | C | A | 0.879 | 0.138 | 1.5E-10 | 0.581 | 446 118 |
|  | rs7915425 | T | C | 1.144 | 0.179 | 2E-10 | 0.175 | 446 118 |
|  | rs330088 | C | T | 0.868 | 0.137 | 2.7E-10 | 0.547 | 446 118 |
|  | rs8038326 | A | G | 0.955 | 0.152 | 2.8E-10 | 0.727 | 446 118 |
|  | rs460692 | C | T | 1.263 | 0.200 | 3.6E-10 | 0.137 | 446 118 |
|  | rs9382445 | T | C | 0.872 | 0.140 | 4.8E-10 | 0.623 | 446 118 |
|  | rs4767550 | G | A | 0.858 | 0.139 | 6.3E-10 | 0.414 | 446 118 |
|  | rs11885663 | T | C | 0.973 | 0.157 | 8.6E-10 | 0.248 | 446 118 |
|  | rs1991556 | G | A | 0.994 | 0.163 | 1.00E-09 | 0.774 | 446 118 |
|  | rs1057703 | G | T | 1.164 | 0.192 | 1.1E-09 | 0.147 | 446 118 |
|  | rs4128364 | C | T | 0.876 | 0.143 | 1.4E-09 | 0.339 | 446 118 |
|  | rs61796569 | T | C | 0.927 | 0.154 | 1.5E-09 | 0.270 | 446 118 |
|  | rs10483350 | G | A | 1.042 | 0.172 | 1.5E-09 | 0.195 | 446 118 |
|  | rs7115226 | A | C | 1.594 | 0.261 | 1.7E-09 | 0.074 | 446 118 |
|  | rs269054 | A | T | 0.819 | 0.138 | 2.1E-09 | 0.422 | 446 118 |
|  | rs112230981 | A | G | 1.892 | 0.314 | 2.2E-09 | 0.950 | 446 118 |
|  | rs11602180 | C | T | 1.095 | 0.184 | 2.3E-09 | 0.837 | 446 118 |
|  | rs2192528 | A | G | 0.802 | 0.136 | 2.7E-09 | 0.480 | 446 118 |
|  | rs12246842 | A | G | 0.804 | 0.136 | 3.9E-09 | 0.460 | 446 118 |
|  | rs205024 | T | C | 0.830 | 0.140 | 3.9E-09 | 0.384 | 446 118 |
|  | rs12567114 | A | G | 0.890 | 0.152 | 4.3E-09 | 0.276 | 446 118 |
|  | rs7616632 | T | G | 0.792 | 0.136 | 4.3E-09 | 0.522 | 446 118 |
|  | rs6575005 | T | C | 0.934 | 0.159 | 4.4E-09 | 0.758 | 446 118 |
|  | rs1776776 | T | C | 1.198 | 0.205 | 4.9E-09 | 0.874 | 446 118 |
|  | rs11621908 | C | T | 1.446 | 0.250 | 5.6E-09 | 0.917 | 446 118 |
|  | rs10421649 | A | T | 0.798 | 0.138 | 6.9E-09 | 0.557 | 446 118 |
|  | rs2072727 | T | C | 0.795 | 0.137 | 7.9E-09 | 0.436 | 446 118 |
|  | rs113113059 | T | C | 0.968 | 0.164 | 8.4E-09 | 0.780 | 446 118 |
|  | rs374153 | C | T | 1.057 | 0.186 | 9.1E-09 | 0.158 | 446 118 |
|  | rs151014368 | A | G | 0.966 | 0.169 | 9.1E-09 | 0.206 | 446 118 |
|  | rs62120041 | T | C | 1.567 | 0.274 | 9.6E-09 | 0.934 | 446 118 |
|  | rs7503199 | C | T | 0.885 | 0.154 | 1.00E-08 | 0.734 | 446 118 |
|  | rs1939455 | G | T | 1.226 | 0.214 | 1.20E-08 | 0.879 | 446 118 |
|  | rs7951019 | G | T | 2.213 | 0.391 | 1.20E-08 | 0.032 | 446 118 |
|  | rs17732997 | C | G | 0.776 | 0.137 | 1.20E-08 | 0.569 | 446 118 |
|  | rs61985058 | T | C | 1.116 | 0.194 | 1.30E-08 | 0.143 | 446 118 |
|  | rs17427571 | A | G | 0.830 | 0.146 | 1.30E-08 | 0.684 | 446 118 |
|  | rs7806045 | T | C | 0.887 | 0.158 | 1.40E-08 | 0.755 | 446 118 |
|  | rs35531607 | C | T | 0.770 | 0.136 | 1.50E-08 | 0.474 | 446 118 |
|  | rs7644809 | T | C | 0.784 | 0.138 | 1.60E-08 | 0.422 | 446 118 |
|  | rs9345234 | C | A | 0.781 | 0.138 | 1.80E-08 | 0.578 | 446 118 |
|  | rs12791153 | T | A | 1.413 | 0.253 | 1.90E-08 | 0.081 | 446 118 |
|  | rs1263056 | A | G | 0.768 | 0.137 | 2.00E-08 | 0.519 | 446 118 |
|  | rs55658675 | C | T | 0.788 | 0.142 | 2.00E-08 | 0.645 | 446 118 |
|  | rs11567976 | T | C | 0.768 | 0.137 | 2.10E-08 | 0.571 | 446 118 |
|  | rs180769 | T | C | 0.763 | 0.138 | 2.30E-08 | 0.425 | 446 118 |
|  | rs1553132 | G | A | 0.870 | 0.155 | 2.50E-08 | 0.258 | 446 118 |
|  | rs9903973 | C | T | 0.766 | 0.136 | 2.60E-08 | 0.467 | 446 118 |
|  | rs11614986 | A | G | 0.983 | 0.177 | 2.70E-08 | 0.821 | 446 118 |
|  | rs2231265 | G | A | 0.897 | 0.162 | 2.70E-08 | 0.772 | 446 118 |
|  | rs174560 | C | T | 0.815 | 0.146 | 2.80E-08 | 0.314 | 446 118 |
|  | rs10173260 | C | T | 0.770 | 0.139 | 2.90E-08 | 0.606 | 446 118 |
|  | rs72804080 | G | A | 1.068 | 0.192 | 2.90E-08 | 0.150 | 446 118 |
|  | rs12611523 | A | G | 0.758 | 0.137 | 3.10E-08 | 0.545 | 446 118 |
|  | rs11643715 | G | C | 0.834 | 0.150 | 3.20E-08 | 0.291 | 446 118 |
|  | rs4538155 | T | C | 0.779 | 0.142 | 3.60E-08 | 0.647 | 446 118 |
|  | rs34354917 | C | A | 0.825 | 0.150 | 3.90E-08 | 0.710 | 446 118 |
|  | rs80193650 | G | A | 1.010 | 0.184 | 4.10E-08 | 0.162 | 446 118 |
|  | rs10761674 | C | T | 0.740 | 0.136 | 4.20E-08 | 0.477 | 446 118 |
|  | rs11190970 | G | A | 0.923 | 0.169 | 4.60E-08 | 0.799 | 446 118 |

Table G3: Summary information of genetic variants identified for short sleep.

| Sr. No. | rsID | Effect Allele | Other Allele | Beta | Standard Error | P | Effect Allele Frequency | N |
| --- | --- | --- | --- | --- | --- | --- | --- | --- |
|  | rs2863957 | C | A | 0.05449 | 0.0072 | 2.60E-18 | 0.782 | 411 934 |
|  | rs13107325 | T | C | 0.07511 | 0.0109 | 2.50E-13 | 0.075 | 411 934 |
|  | rs1229762 | T | C | 0.03730 | 0.0064 | 1.10E-12 | 0.665 | 411 934 |
|  | rs1380703 | G | A | 0.03537 | 0.0062 | 1.60E-11 | 0.384 | 411 934 |
|  | rs12963463 | C | T | 0.02859 | 0.0064 | 1.90E-11 | 0.299 | 411 934 |
|  | rs75539574 | A | C | 0.04497 | 0.0107 | 8.40E-11 | 0.915 | 411 934 |
|  | rs17388803 | C | A | 0.05259 | 0.0097 | 6.50E-10 | 0.106 | 411 934 |
|  | rs4585442 | G | A | 0.03053 | 0.0062 | 8.10E-10 | 0.311 | 411 934 |
|  | rs1607227 | G | T | 0.03053 | 0.0067 | 1.50E-09 | 0.705 | 411 934 |
|  | rs2820313 | G | A | 0.03053 | 0.0059 | 2.30E-09 | 0.341 | 411 934 |
|  | rs17005118 | A | G | 0.02956 | 0.0067 | 2.50E-09 | 0.265 | 411 934 |
|  | rs5757675 | G | T | 0.03440 | 0.0067 | 2.70E-09 | 0.260 | 411 934 |
|  | rs12567114 | G | A | 0.03633 | 0.0066 | 4.10E-09 | 0.725 | 411 934 |
|  | rs142180737 | C | T | 0.15444 | 0.0317 | 4.40E-09 | 0.009 | 411 934 |
|  | rs2186122 | T | A | 0.02372 | 0.0060 | 4.80E-09 | 0.562 | 411 934 |
|  | rs11763750 | G | A | 0.03537 | 0.0076 | 5.10E-09 | 0.814 | 411 934 |
|  | rs12518468 | C | T | 0.03150 | 0.0062 | 8.50E-09 | 0.328 | 411 934 |
|  | rs9367621 | T | A | 0.02372 | 0.0057 | 1.60E-08 | 0.431 | 411 934 |
|  | rs3776864 | A | C | 0.03150 | 0.0064 | 1.70E-08 | 0.667 | 411 934 |
|  | rs60882754 | A | T | 0.05543 | 0.0123 | 1.80E-08 | 0.939 | 411 934 |
|  | rs59779556 | T | G | 0.02469 | 0.0060 | 2.00E-08 | 0.554 | 411 934 |
|  | rs2014830 | C | T | 0.02956 | 0.0064 | 2.70E-08 | 0.698 | 411 934 |
|  | rs205024 | C | T | 0.03053 | 0.0062 | 2.70E-08 | 0.617 | 411 934 |
|  | rs12661667 | T | C | 0.02762 | 0.0067 | 2.80E-08 | 0.263 | 411 934 |
|  | rs7939345 | T | G | 0.03537 | 0.0071 | 4.00E-08 | 0.208 | 411 934 |
|  | rs9321171 | C | T | 0.03150 | 0.0059 | 4.20E-08 | 0.540 | 411 934 |
|  | rs7524118 | C | T | 0.02956 | 0.0064 | 4.90E-08 | 0.708 | 411 934 |

Table G4: Summary information of genetic variants identified for long sleep.

| Sr. No. | rsID | Effect Allele | Other Allele | Beta | Standard Error | P | Effect Allele Frequency | N |
| --- | --- | --- | --- | --- | --- | --- | --- | --- |
|  | rs6737318 | G | A | 0.07603 | 0.0111 | 3.40E-13 | 0.222 | 339 926 |
|  | rs75458655 | T | C | 0.18482 | 0.0294 | 5.40E-12 | 0.023 | 339 926 |
|  | rs17688916 | T | A | 0.07139 | 0.0124 | 1.10E-11 | 0.796 | 339 926 |
|  | rs17817288 | A | G | 0.03922 | 0.0093 | 8.90E-09 | 0.518 | 339 926 |
|  | rs549961083 | T | C | 0.53357 | 0.1166 | 9.60E-09 | 0.001 | 339 926 |
|  | rs3751046 | G | A | 0.06953 | 0.0131 | 2.00E-08 | 0.147 | 339 926 |
|  | rs7534398 | A | T | 0.04688 | 0.0117 | 2.10E-08 | 0.201 | 339 926 |
|  | rs10899257 | A | G | 0.06766 | 0.0131 | 4.60E-08 | 0.144 | 339 926 |

Table G5: Summary information of genetic variants identified for chronotype.

| Sr. No. | rsID | Effect Allele | Other Allele | Beta | Standard Error | P | Effect Allele Frequency | N | Replication in 23andMe |
| --- | --- | --- | --- | --- | --- | --- | --- | --- | --- |
|  | rs909757 | T | C | 0.0197 | 0.003283 | 1.96E-09 | 0.63 | 651 295 | No |
|  | rs61773390 | T | G | 0.0659 | 0.004687 | 6.55E-45 | 0.1931 | 651 295 | Yes |
|  | rs12065331 | T | C | -0.0244 | 0.003462 | 1.82E-12 | 0.3125 | 651 295 | No |
|  | rs17448682 | T | C | 0.0348 | 0.004059 | 9.97E-18 | 0.2339 | 651 295 | No |
|  | rs10917513 | T | C | -0.0309 | 0.003739 | 1.41E-16 | 0.6476 | 651 295 | Yes |
|  | rs10916892 | T | C | -0.0345 | 0.003695 | 9.90E-21 | 0.6203 | 651 295 | Yes |
|  | rs2506089 | T | G | 0.0198 | 0.003403 | 5.95E-09 | 0.5682 | 651 295 | No |
|  | rs12140153 | T | G | -0.0604 | 0.006493 | 1.36E-20 | 0.0892 | 651 295 | Yes |
|  | rs11208844 | A | G | -0.029 | 0.004742 | 9.61E-10 | 0.1408 | 651 295 | No |
|  | rs12040629 | A | G | 0.0727 | 0.004925 | 2.57E-49 | 0.16 | 651 295 | Yes |
|  | rs11588913 | A | G | -0.0236 | 0.003576 | 4.11E-11 | 0.4037 | 651 295 | No |
|  | rs5016898 | T | C | -0.0241 | 0.003694 | 6.83E-11 | 0.4238 | 651 295 | No |
|  | rs72720396 | A | G | -0.0416 | 0.004203 | 4.26E-23 | 0.7741 | 651 295 | No |
|  | rs481214 | A | T | 0.0232 | 0.003948 | 4.17E-09 | 0.6063 | 651 295 | No |
|  | rs11165655 | A | G | -0.0281 | 0.003708 | 3.48E-14 | 0.5281 | 651 295 | No |
|  | rs17575798 | A | G | -0.0336 | 0.004233 | 2.08E-15 | 0.1918 | 651 295 | No |
|  | rs6690292 | T | C | -0.0247 | 0.004165 | 3.04E-09 | 0.7285 | 651 295 | No |
|  | rs11102807 | A | G | -0.0219 | 0.003623 | 1.50E-09 | 0.5361 | 651 295 | No |
|  | rs9436119 | A | G | 0.0395 | 0.003314 | 9.31E-33 | 0.3842 | 651 295 | Yes |
|  | rs6665637 | A | G | -0.0196 | 0.003107 | 2.80E-10 | 0.2804 | 651 295 | No |
|  | rs115073088 | A | G | -0.0755 | 0.010399 | 3.88E-13 | 0.975 | 651 295 | No |
|  | rs975025 | T | C | -0.049 | 0.006244 | 4.21E-15 | 0.0758 | 651 295 | No |
|  | rs1144566 | T | C | 0.2306 | 0.010615 | 1.24E-104 | 0.029 | 651 295 | Yes |
|  | rs1221502 | A | C | 0.0197 | 0.00331 | 2.67E-09 | 0.7386 | 651 295 | No |
|  | rs4657983 | A | G | -0.0252 | 0.003698 | 9.45E-12 | 0.6511 | 651 295 | No |
|  | rs6429233 | A | G | 0.0202 | 0.003382 | 2.33E-09 | 0.4574 | 651 295 | No |
|  | rs13011556 | C | G | -0.0285 | 0.003663 | 7.21E-15 | 0.7624 | 651 295 | No |
|  | rs62124718 | A | G | -0.0447 | 0.005831 | 1.78E-14 | 0.895 | 651 295 | Yes |
|  | rs72796401 | A | T | 0.0246 | 0.003266 | 5.02E-14 | 0.1903 | 651 295 | No |
|  | rs6718511 | A | G | 0.0209 | 0.003483 | 1.96E-09 | 0.557 | 651 295 | No |
|  | rs11678584 | A | T | -0.0278 | 0.004512 | 7.24E-10 | 0.8599 | 651 295 | No |
|  | rs848552 | C | G | -0.0282 | 0.003571 | 2.86E-15 | 0.4755 | 651 295 | No |
|  | rs7602499 | T | C | 0.0223 | 0.003861 | 7.63E-09 | 0.3536 | 651 295 | No |
|  | rs75120545 | T | C | 0.0862 | 0.00985 | 2.11E-18 | 0.0319 | 651 295 | No |
|  | rs6544906 | A | C | 0.0233 | 0.003588 | 8.38E-11 | 0.5616 | 651 295 | No |
|  | rs17396357 | T | C | 0.0211 | 0.003602 | 4.68E-09 | 0.3818 | 651 295 | No |
|  | rs12470914 | A | T | 0.053 | 0.005982 | 8.00E-19 | 0.1005 | 651 295 | Yes |
|  | rs4672458 | T | C | -0.0224 | 0.003345 | 2.13E-11 | 0.4764 | 651 295 | No |
|  | rs13414393 | T | C | -0.0219 | 0.003595 | 1.12E-09 | 0.5405 | 651 295 | No |
|  | rs10175975 | T | C | 0.0252 | 0.003946 | 1.70E-10 | 0.1853 | 651 295 | No |
|  | rs359248 | T | G | -0.0275 | 0.003196 | 7.69E-18 | 0.4564 | 651 295 | Yes |
|  | rs812925 | C | G | -0.0306 | 0.003496 | 2.06E-18 | 0.6458 | 651 295 | No |
|  | rs113851554 | T | G | -0.054 | 0.006988 | 1.09E-14 | 0.0567 | 651 295 | No |
|  | rs2706762 | T | C | -0.037 | 0.004661 | 2.05E-15 | 0.1496 | 651 295 | No |
|  | rs12464387 | A | G | -0.0214 | 0.003347 | 1.62E-10 | 0.4638 | 651 295 | No |
|  | rs6727752 | A | G | 0.0256 | 0.003851 | 2.99E-11 | 0.3569 | 651 295 | No |
|  | rs10520176 | T | C | 0.0377 | 0.003555 | 2.80E-26 | 0.4926 | 651 295 | Yes |
|  | rs11681299 | T | C | 0.0237 | 0.003887 | 1.08E-09 | 0.2835 | 651 295 | No |
|  | rs34509802 | A | G | 0.0396 | 0.005328 | 1.07E-13 | 0.1785 | 651 295 | Yes |
|  | rs76064513 | T | C | 0.0338 | 0.005825 | 6.53E-09 | 0.1342 | 651 295 | No |
|  | rs77248969 | A | G | -0.0333 | 0.005431 | 8.69E-10 | 0.1116 | 651 295 | No |
|  | rs28380327 | A | T | 0.04 | 0.003695 | 2.61E-27 | 0.6317 | 651 295 | Yes |
|  | rs2166559 | T | C | -0.0329 | 0.005198 | 2.47E-10 | 0.8611 | 651 295 | No |
|  | rs747003 | T | C | 0.0196 | 0.003367 | 5.83E-09 | 0.6088 | 651 295 | No |
|  | rs13004345 | T | C | -0.0189 | 0.003014 | 3.62E-10 | 0.6475 | 651 295 | No |
|  | rs6433478 | T | C | -0.0253 | 0.003558 | 1.16E-12 | 0.4601 | 651 295 | Yes |
|  | rs4666682 | A | G | -0.0254 | 0.004109 | 6.34E-10 | 0.1797 | 651 295 | No |
|  | rs11677484 | T | G | 0.0229 | 0.003592 | 1.82E-10 | 0.2574 | 651 295 | No |
|  | rs1064213 | A | G | 0.0444 | 0.00365 | 4.78E-34 | 0.4826 | 651 295 | Yes |
|  | rs184033703 | A | G | 0.0577 | 0.007435 | 8.41E-15 | 0.0585 | 651 295 | No |
|  | rs80271258 | T | C | -0.0894 | 0.006197 | 3.52E-47 | 0.0844 | 651 295 | Yes |
|  | rs62182135 | A | C | -0.0242 | 0.003246 | 8.97E-14 | 0.3285 | 651 295 | No |
|  | rs35346733 | A | G | -0.0316 | 0.004832 | 6.14E-11 | 0.1942 | 651 295 | No |
|  | rs111261826 | A | C | -0.028 | 0.004043 | 4.32E-12 | 0.679 | 651 295 | No |
|  | rs149611468 | T | C | 0.1432 | 0.017515 | 2.94E-16 | 0.9888 | 651 295 | No |
|  | rs6794796 | A | G | 0.0253 | 0.003834 | 4.16E-11 | 0.2897 | 651 295 | No |
|  | rs9817910 | A | G | -0.0216 | 0.003231 | 2.30E-11 | 0.5576 | 651 295 | No |
|  | rs73050286 | T | C | 0.0297 | 0.004278 | 3.88E-12 | 0.7817 | 651 295 | No |
|  | rs2362775 | T | C | -0.0218 | 0.003576 | 1.09E-09 | 0.5342 | 651 295 | No |
|  | rs114848860 | A | T | -0.0768 | 0.009878 | 7.56E-15 | 0.9737 | 651 295 | No |
|  | rs78580841 | T | C | 0.0424 | 0.006106 | 3.81E-12 | 0.0694 | 651 295 | No |
|  | rs12636669 | T | C | 0.057 | 0.006106 | 1.01E-20 | 0.0801 | 651 295 | Yes |
|  | rs17007397 | C | G | 0.0231 | 0.004036 | 1.05E-08 | 0.5801 | 651 295 | No |
|  | rs7626335 | A | C | -0.0286 | 0.003965 | 5.44E-13 | 0.3305 | 651 295 | Yes |
|  | rs7429614 | T | G | 0.0349 | 0.003703 | 4.36E-21 | 0.416 | 651 295 | Yes |
|  | rs12631477 | T | C | 0.0277 | 0.004463 | 5.42E-10 | 0.7982 | 651 295 | No |
|  | rs1449403 | A | G | 0.0424 | 0.005626 | 4.81E-14 | 0.1226 | 651 295 | Yes |
|  | rs34967119 | A | G | 0.02 | 0.003337 | 2.04E-09 | 0.4958 | 651 295 | No |
|  | rs1398346 | T | C | 0.0259 | 0.004513 | 9.51E-09 | 0.8668 | 651 295 | No |
|  | rs1800828 | C | G | 0.0257 | 0.00437 | 4.09E-09 | 0.7508 | 651 295 | No |
|  | rs72950188 | T | C | 0.0447 | 0.006676 | 2.14E-11 | 0.9238 | 651 295 | No |
|  | rs72966564 | T | C | -0.0234 | 0.003861 | 1.36E-09 | 0.2507 | 651 295 | No |
|  | rs13065394 | T | G | -0.0274 | 0.003962 | 4.64E-12 | 0.286 | 651 295 | No |
|  | rs4550782 | T | G | 0.0282 | 0.003897 | 4.60E-13 | 0.6655 | 651 295 | No |
|  | rs7649164 | T | G | 0.0211 | 0.003466 | 1.15E-09 | 0.5745 | 651 295 | No |
|  | rs6440833 | A | G | 0.021 | 0.00327 | 1.35E-10 | 0.4638 | 651 295 | No |
|  | rs111867612 | A | C | -0.032 | 0.005572 | 9.30E-09 | 0.1021 | 651 295 | No |
|  | rs1599374 | A | G | 0.0309 | 0.003738 | 1.38E-16 | 0.5163 | 651 295 | Yes |
|  | rs3850174 | A | T | -0.0346 | 0.004181 | 1.28E-16 | 0.2575 | 651 295 | Yes |
|  | rs301218 | A | G | -0.0237 | 0.003527 | 1.81E-11 | 0.3916 | 651 295 | No |
|  | rs9836621 | T | C | -0.0279 | 0.003617 | 1.22E-14 | 0.5217 | 651 295 | Yes |
|  | rs1468945 | A | G | -0.0363 | 0.00428 | 2.21E-17 | 0.7852 | 651 295 | No |
|  | rs3796618 | A | T | -0.0229 | 0.00381 | 1.85E-09 | 0.5293 | 651 295 | No |
|  | rs4690085 | A | G | -0.0193 | 0.003282 | 4.10E-09 | 0.5348 | 651 295 | No |
|  | rs4698678 | C | G | 0.0307 | 0.004324 | 1.24E-12 | 0.2786 | 651 295 | Yes |
|  | rs1502249 | A | G | 0.0173 | 0.002969 | 5.67E-09 | 0.5223 | 651 295 | No |
|  | rs6838677 | A | C | -0.0213 | 0.003654 | 5.58E-09 | 0.6689 | 651 295 | No |
|  | rs4860734 | A | G | 0.0195 | 0.003345 | 5.55E-09 | 0.2897 | 651 295 | No |
|  | rs6816922 | A | C | -0.02 | 0.003483 | 9.30E-09 | 0.5377 | 651 295 | No |
|  | rs6846730 | T | C | -0.0323 | 0.004045 | 1.39E-15 | 0.2353 | 651 295 | No |
|  | rs2850979 | T | C | -0.0234 | 0.003771 | 5.45E-10 | 0.7603 | 651 295 | No |
|  | rs7700110 | A | G | 0.0242 | 0.003927 | 7.16E-10 | 0.2566 | 651 295 | No |
|  | rs17455138 | T | C | 0.0307 | 0.004533 | 1.27E-11 | 0.7655 | 651 295 | Yes |
|  | rs4241964 | T | G | -0.0287 | 0.003405 | 3.45E-17 | 0.5217 | 651 295 | No |
|  | rs938836 | A | G | -0.0212 | 0.003311 | 1.53E-10 | 0.467 | 651 295 | No |
|  | rs72729847 | T | C | -0.0303 | 0.004502 | 1.69E-11 | 0.8005 | 651 295 | No |
|  | rs9997394 | A | G | -0.0254 | 0.003971 | 1.60E-10 | 0.2866 | 651 295 | No |
|  | rs10058356 | T | C | -0.0207 | 0.003294 | 3.28E-10 | 0.6958 | 651 295 | No |
|  | rs7701529 | A | T | -0.0295 | 0.004077 | 4.67E-13 | 0.2393 | 651 295 | No |
|  | rs7721608 | T | G | 0.0204 | 0.003246 | 3.27E-10 | 0.4648 | 651 295 | No |
|  | rs66507804 | T | C | -0.0322 | 0.00465 | 4.38E-12 | 0.799 | 651 295 | No |
|  | rs4269995 | T | C | -0.0339 | 0.00354 | 1.01E-21 | 0.251 | 651 295 | Yes |
|  | rs77960 | A | G | 0.0221 | 0.003246 | 9.92E-12 | 0.3304 | 651 295 | No |
|  | rs1559253 | A | G | 0.0217 | 0.00355 | 9.82E-10 | 0.3588 | 651 295 | Yes |
|  | rs17140201 | A | G | -0.0285 | 0.004745 | 1.90E-09 | 0.172 | 651 295 | No |
|  | rs13172141 | A | T | 0.0217 | 0.003443 | 2.94E-10 | 0.5694 | 651 295 | No |
|  | rs67988891 | C | G | -0.0363 | 0.003945 | 3.50E-20 | 0.6821 | 651 295 | No |
|  | rs2901796 | A | G | 0.0246 | 0.003792 | 8.79E-11 | 0.3983 | 651 295 | No |
|  | rs42210 | C | G | -0.0291 | 0.004463 | 6.97E-11 | 0.7124 | 651 295 | No |
|  | rs12518401 | A | G | -0.0236 | 0.00352 | 2.01E-11 | 0.3844 | 651 295 | No |
|  | rs7735794 | A | G | 0.0344 | 0.005873 | 4.70E-09 | 0.2241 | 651 295 | No |
|  | rs465670 | T | C | 0.0241 | 0.003643 | 3.68E-11 | 0.5404 | 651 295 | No |
|  | rs9394154 | C | G | -0.0217 | 0.003373 | 1.25E-10 | 0.436 | 651 295 | No |
|  | rs9381812 | A | G | -0.0495 | 0.003905 | 8.15E-37 | 0.7051 | 651 295 | Yes |
|  | rs1811899 | T | C | -0.0298 | 0.004688 | 2.06E-10 | 0.7895 | 651 295 | No |
|  | rs9465253 | T | C | 0.023 | 0.0037 | 5.07E-10 | 0.2801 | 651 295 | No |
|  | rs766406 | T | G | -0.0236 | 0.003652 | 1.03E-10 | 0.6409 | 651 295 | Yes |
|  | rs486416 | A | G | -0.0201 | 0.003166 | 2.18E-10 | 0.6527 | 651 295 | No |
|  | rs13203140 | T | C | -0.0252 | 0.003985 | 2.56E-10 | 0.6353 | 651 295 | No |
|  | rs3923809 | A | G | -0.022 | 0.003792 | 6.55E-09 | 0.6947 | 651 295 | No |
|  | rs12206814 | C | G | 0.0254 | 0.004249 | 2.26E-09 | 0.4869 | 651 295 | No |
|  | rs2396004 | A | G | 0.0214 | 0.003443 | 5.14E-10 | 0.4354 | 651 295 | No |
|  | rs3857599 | A | C | 0.0322 | 0.005142 | 3.81E-10 | 0.1659 | 651 295 | No |
|  | rs2653349 | A | G | 0.0659 | 0.004304 | 6.58E-53 | 0.2073 | 651 295 | Yes |
|  | rs9476310 | T | C | 0.0263 | 0.003502 | 5.90E-14 | 0.5104 | 651 295 | No |
|  | rs1931814 | A | G | 0.0264 | 0.003453 | 2.08E-14 | 0.4787 | 651 295 | No |
|  | rs2881955 | T | C | 0.0271 | 0.003939 | 5.99E-12 | 0.2788 | 651 295 | No |
|  | rs12195792 | A | T | 0.034 | 0.004142 | 2.24E-16 | 0.2717 | 651 295 | Yes |
|  | rs11154718 | T | C | -0.0233 | 0.003672 | 2.20E-10 | 0.43 | 651 295 | No |
|  | rs60616179 | A | G | 0.0509 | 0.00772 | 4.31E-11 | 0.9443 | 651 295 | No |
|  | rs4535583 | T | C | 0.0212 | 0.003674 | 7.92E-09 | 0.6991 | 651 295 | No |
|  | rs9496623 | A | G | -0.0235 | 0.004025 | 5.27E-09 | 0.7328 | 651 295 | No |
|  | rs2050185 | A | G | 0.0217 | 0.00357 | 1.21E-09 | 0.6245 | 651 295 | No |
|  | rs9479402 | T | C | -0.2186 | 0.017396 | 3.26E-36 | 0.9883 | 651 295 | Yes |
|  | rs9347926 | A | T | 0.0264 | 0.0034 | 8.16E-15 | 0.4407 | 651 295 | No |
|  | rs9348050 | T | C | 0.0221 | 0.003233 | 8.19E-12 | 0.4864 | 651 295 | No |
|  | rs4027217 | A | C | -0.0258 | 0.004499 | 9.76E-09 | 0.217 | 651 295 | No |
|  | rs10237162 | T | C | 0.0368 | 0.004006 | 4.04E-20 | 0.7232 | 651 295 | Yes |
|  | rs10951325 | T | C | 0.0336 | 0.003517 | 1.24E-21 | 0.6319 | 651 295 | Yes |
|  | rs6967481 | T | C | 0.0321 | 0.003274 | 1.08E-22 | 0.5011 | 651 295 | Yes |
|  | rs4236237 | A | C | -0.0242 | 0.003574 | 1.27E-11 | 0.5992 | 651 295 | No |
|  | rs2944831 | A | G | 0.025 | 0.003674 | 1.02E-11 | 0.2946 | 651 295 | No |
|  | rs3807651 | A | T | 0.0246 | 0.00408 | 1.64E-09 | 0.4938 | 651 295 | No |
|  | rs10254050 | C | G | -0.0577 | 0.00491 | 7.01E-32 | 0.1905 | 651 295 | Yes |
|  | rs4729854 | A | T | -0.0485 | 0.003689 | 1.76E-39 | 0.4701 | 651 295 | Yes |
|  | rs2396719 | A | G | 0.0331 | 0.004431 | 8.04E-14 | 0.239 | 651 295 | No |
|  | rs17302081 | T | C | 0.022 | 0.003642 | 1.53E-09 | 0.4414 | 651 295 | No |
|  | rs6968240 | A | C | 0.0216 | 0.003262 | 3.54E-11 | 0.4171 | 651 295 | No |
|  | rs62465218 | A | C | -0.0274 | 0.004558 | 1.84E-09 | 0.1456 | 651 295 | No |
|  | rs6958557 | T | G | 0.0261 | 0.003591 | 3.62E-13 | 0.6067 | 651 295 | Yes |
|  | rs113161209 | A | G | 0.0442 | 0.007651 | 7.59E-09 | 0.077 | 651 295 | No |
|  | rs2072413 | T | C | -0.0208 | 0.00364 | 1.11E-08 | 0.2624 | 651 295 | No |
|  | rs62479736 | T | G | 0.0243 | 0.003709 | 5.73E-11 | 0.2915 | 651 295 | No |
|  | rs35524253 | A | G | 0.0342 | 0.003749 | 7.30E-20 | 0.3539 | 651 295 | Yes |
|  | rs2979139 | A | G | -0.0267 | 0.003368 | 2.24E-15 | 0.5048 | 651 295 | No |
|  | rs2322605 | A | G | -0.0215 | 0.003438 | 4.03E-10 | 0.4684 | 651 295 | No |
|  | rs71523448 | C | G | -0.0499 | 0.006454 | 1.06E-14 | 0.0788 | 651 295 | No |
|  | rs6993892 | T | C | -0.0347 | 0.003615 | 8.04E-22 | 0.6162 | 651 295 | Yes |
|  | rs6468316 | T | C | -0.0197 | 0.00339 | 6.22E-09 | 0.4708 | 651 295 | No |
|  | rs7845620 | A | C | -0.0425 | 0.004577 | 1.62E-20 | 0.8344 | 651 295 | Yes |
|  | rs10109566 | A | G | -0.0222 | 0.00368 | 1.62E-09 | 0.4849 | 651 295 | No |
|  | rs34054660 | A | G | 0.0249 | 0.004343 | 9.82E-09 | 0.5747 | 651 295 | Yes |
|  | rs187028 | A | T | -0.0222 | 0.003336 | 2.83E-11 | 0.3146 | 651 295 | No |
|  | rs16939162 | A | G | 0.038 | 0.004701 | 6.26E-16 | 0.8304 | 651 295 | No |
|  | rs6988733 | T | C | 0.0229 | 0.003934 | 5.84E-09 | 0.3491 | 651 295 | No |
|  | rs7006885 | A | G | 0.0301 | 0.004209 | 8.56E-13 | 0.2878 | 651 295 | Yes |
|  | rs3100052 | A | G | 0.0246 | 0.003525 | 2.99E-12 | 0.3886 | 651 295 | No |
|  | rs2737245 | T | G | 0.0335 | 0.003778 | 7.56E-19 | 0.2748 | 651 295 | No |
|  | rs1871729 | A | G | -0.0232 | 0.003834 | 1.44E-09 | 0.6816 | 651 295 | No |
|  | rs6477309 | T | C | 0.0309 | 0.003762 | 2.16E-16 | 0.6661 | 651 295 | Yes |
|  | rs2844016 | T | C | 0.0266 | 0.004253 | 3.99E-10 | 0.2935 | 651 295 | No |
|  | rs308521 | T | C | 0.0283 | 0.003206 | 1.06E-18 | 0.6039 | 651 295 | No |
|  | rs4878734 | A | T | 0.0218 | 0.003808 | 1.03E-08 | 0.5114 | 651 295 | No |
|  | rs6560218 | T | C | -0.0224 | 0.003766 | 2.72E-09 | 0.5183 | 651 295 | No |
|  | rs62553781 | T | C | -0.0689 | 0.009233 | 8.49E-14 | 0.0329 | 651 295 | No |
|  | rs12378543 | T | C | -0.0233 | 0.003797 | 8.43E-10 | 0.3841 | 651 295 | No |
|  | rs555784 | A | T | -0.0249 | 0.003777 | 4.33E-11 | 0.3803 | 651 295 | No |
|  | rs295268 | T | C | -0.0308 | 0.004596 | 2.05E-11 | 0.7403 | 651 295 | No |
|  | rs3138490 | A | T | 0.0235 | 0.003636 | 1.03E-10 | 0.515 | 651 295 | No |
|  | rs10759208 | T | C | -0.0248 | 0.003793 | 6.22E-11 | 0.6138 | 651 295 | No |
|  | rs11788633 | C | G | 0.0197 | 0.003247 | 1.30E-09 | 0.6547 | 651 295 | No |
|  | rs10818834 | T | C | 0.0302 | 0.004168 | 4.30E-13 | 0.7297 | 651 295 | No |
|  | rs10988239 | T | C | -0.0213 | 0.003063 | 3.56E-12 | 0.5117 | 651 295 | No |
|  | rs12380242 | T | C | -0.0206 | 0.003194 | 1.12E-10 | 0.5058 | 651 295 | No |
|  | rs28458909 | T | C | -0.0702 | 0.005531 | 6.65E-37 | 0.1225 | 651 295 | Yes |
|  | rs497338 | T | C | 0.0271 | 0.00406 | 2.48E-11 | 0.2881 | 651 295 | No |
|  | rs66617308 | T | C | 0.0184 | 0.003207 | 9.62E-09 | 0.6713 | 651 295 | No |
|  | rs9416744 | A | C | 0.0344 | 0.003935 | 2.29E-18 | 0.2595 | 651 295 | Yes |
|  | rs11597421 | A | G | -0.0238 | 0.004032 | 3.57E-09 | 0.4973 | 651 295 | No |
|  | rs12249410 | T | G | -0.0337 | 0.005395 | 4.22E-10 | 0.1077 | 651 295 | No |
|  | rs17712705 | A | G | -0.0247 | 0.003989 | 5.94E-10 | 0.3265 | 651 295 | No |
|  | rs2298117 | T | C | -0.0225 | 0.003653 | 7.31E-10 | 0.448 | 651 295 | No |
|  | rs10762434 | C | G | 0.0249 | 0.004246 | 4.50E-09 | 0.7752 | 651 295 | No |
|  | rs2648721 | T | G | -0.0238 | 0.004061 | 4.60E-09 | 0.7039 | 651 295 | No |
|  | rs61875203 | T | C | 0.0259 | 0.004118 | 3.19E-10 | 0.2773 | 651 295 | No |
|  | rs1163238 | A | G | -0.0237 | 0.004099 | 7.36E-09 | 0.3885 | 651 295 | No |
|  | rs7900191 | T | C | -0.0187 | 0.003269 | 1.07E-08 | 0.4035 | 651 295 | No |
|  | rs11200159 | A | C | -0.0234 | 0.003798 | 7.23E-10 | 0.6551 | 651 295 | No |
|  | rs3808964 | T | G | 0.02 | 0.00336 | 2.63E-09 | 0.6326 | 651 295 | No |
|  | rs9664044 | T | C | -0.0268 | 0.004263 | 3.26E-10 | 0.2319 | 651 295 | No |
|  | rs10830107 | A | G | 0.0283 | 0.004606 | 8.07E-10 | 0.7922 | 651 295 | No |
|  | rs76518095 | T | C | 0.0398 | 0.006579 | 1.45E-09 | 0.0772 | 651 295 | No |
|  | rs12771973 | A | G | -0.0223 | 0.0038 | 4.41E-09 | 0.2475 | 651 295 | No |
|  | rs10832648 | A | C | -0.0314 | 0.004374 | 7.03E-13 | 0.1989 | 651 295 | No |
|  | rs10742179 | A | G | 0.0352 | 0.004264 | 1.52E-16 | 0.2627 | 651 295 | Yes |
|  | rs4923541 | T | C | 0.0247 | 0.003807 | 8.69E-11 | 0.5104 | 651 295 | No |
|  | rs621421 | T | C | -0.0273 | 0.003409 | 1.17E-15 | 0.6256 | 651 295 | No |
|  | rs11032362 | A | G | 0.0704 | 0.006162 | 3.17E-30 | 0.0937 | 651 295 | Yes |
|  | rs7111582 | A | G | -0.0388 | 0.004898 | 2.36E-15 | 0.8956 | 651 295 | No |
|  | rs10838687 | T | G | 0.0345 | 0.004475 | 1.26E-14 | 0.7918 | 651 295 | Yes |
|  | rs12808544 | A | C | -0.0348 | 0.00421 | 1.37E-16 | 0.2409 | 651 295 | No |
|  | rs662094 | A | G | 0.0282 | 0.00373 | 4.01E-14 | 0.4944 | 651 295 | Yes |
|  | rs1278402 | A | G | 0.0281 | 0.004492 | 3.96E-10 | 0.736 | 651 295 | Yes |
|  | rs1508608 | A | G | 0.028 | 0.003734 | 6.42E-14 | 0.3199 | 651 295 | Yes |
|  | rs4121878 | C | G | 0.0219 | 0.003628 | 1.57E-09 | 0.5008 | 651 295 | No |
|  | rs17577073 | A | C | 0.0246 | 0.003691 | 2.64E-11 | 0.5636 | 651 295 | No |
|  | rs2514214 | A | G | 0.027 | 0.004294 | 3.22E-10 | 0.2691 | 651 295 | No |
|  | rs4936290 | A | C | -0.0229 | 0.0034 | 1.64E-11 | 0.6584 | 651 295 | No |
|  | rs3867239 | A | G | 0.0262 | 0.003725 | 2.02E-12 | 0.3762 | 651 295 | No |
|  | rs74357745 | A | G | 0.0312 | 0.004756 | 5.39E-11 | 0.8803 | 651 295 | No |
|  | rs7943634 | T | C | -0.0235 | 0.003655 | 1.28E-10 | 0.3075 | 651 295 | No |
|  | rs3782860 | T | C | 0.025 | 0.003646 | 7.05E-12 | 0.543 | 651 295 | No |
|  | rs1799464 | A | G | -0.02 | 0.003439 | 6.01E-09 | 0.2868 | 651 295 | No |
|  | rs12298405 | T | C | -0.0234 | 0.003495 | 2.16E-11 | 0.3301 | 651 295 | No |
|  | rs2433634 | A | C | -0.0268 | 0.004102 | 6.44E-11 | 0.7241 | 651 295 | No |
|  | rs11611435 | T | C | 0.0278 | 0.004063 | 7.75E-12 | 0.5561 | 651 295 | No |
|  | rs13377754 | T | C | 0.0485 | 0.003484 | 4.85E-44 | 0.6104 | 651 295 | Yes |
|  | rs1843888 | A | G | 0.0513 | 0.003546 | 1.89E-47 | 0.5433 | 651 295 | Yes |
|  | rs247929 | C | G | 0.031 | 0.003526 | 1.47E-18 | 0.5081 | 651 295 | Yes |
|  | rs7975791 | T | C | 0.0513 | 0.008373 | 8.94E-10 | 0.0384 | 651 295 | No |
|  | rs4761989 | T | C | -0.0333 | 0.005161 | 1.11E-10 | 0.868 | 651 295 | No |
|  | rs7299922 | A | G | 0.0239 | 0.003476 | 6.17E-12 | 0.6388 | 651 295 | No |
|  | rs487722 | T | G | 0.0273 | 0.004331 | 2.91E-10 | 0.2084 | 651 295 | No |
|  | rs10877962 | T | C | 0.0359 | 0.003943 | 8.59E-20 | 0.4083 | 651 295 | Yes |
|  | rs711098 | A | C | 0.0217 | 0.00356 | 1.10E-09 | 0.3979 | 651 295 | Yes |
|  | rs7959983 | T | C | -0.0299 | 0.003512 | 1.68E-17 | 0.5955 | 651 295 | No |
|  | rs7304278 | A | G | -0.0291 | 0.003613 | 7.97E-16 | 0.2827 | 651 295 | No |
|  | rs7298532 | T | C | 0.027 | 0.004016 | 1.79E-11 | 0.7213 | 651 295 | No |
|  | rs3955311 | T | C | 0.026 | 0.004464 | 5.71E-09 | 0.1491 | 651 295 | No |
|  | rs80097534 | T | G | -0.0357 | 0.00573 | 4.66E-10 | 0.0966 | 651 295 | No |
|  | rs9597241 | A | C | 0.0332 | 0.004252 | 5.83E-15 | 0.8106 | 651 295 | No |
|  | rs12871550 | A | G | 0.0268 | 0.003612 | 1.18E-13 | 0.3229 | 651 295 | No |
|  | rs9571526 | T | G | -0.0272 | 0.004442 | 9.15E-10 | 0.7706 | 651 295 | No |
|  | rs2593487 | A | G | -0.0285 | 0.003867 | 1.70E-13 | 0.3361 | 651 295 | No |
|  | rs495593 | A | G | 0.0226 | 0.003656 | 6.32E-10 | 0.7383 | 651 295 | No |
|  | rs45597035 | A | G | -0.0222 | 0.003774 | 4.06E-09 | 0.6469 | 651 295 | No |
|  | rs9573980 | A | G | 0.1265 | 0.009631 | 2.10E-39 | 0.9659 | 651 295 | Yes |
|  | rs1886205 | A | C | 0.0293 | 0.003726 | 3.73E-15 | 0.7604 | 651 295 | No |
|  | rs9558942 | T | C | -0.0192 | 0.003347 | 9.70E-09 | 0.6722 | 651 295 | No |
|  | rs3815983 | T | C | -0.0216 | 0.003299 | 5.83E-11 | 0.3604 | 651 295 | No |
|  | rs1163628 | A | C | -0.0287 | 0.004639 | 6.14E-10 | 0.8572 | 651 295 | No |
|  | rs61990287 | A | C | 0.0248 | 0.004068 | 1.09E-09 | 0.2742 | 651 295 | No |
|  | rs2878172 | A | G | -0.0213 | 0.003508 | 1.27E-09 | 0.5693 | 651 295 | No |
|  | rs962961 | T | C | -0.0219 | 0.003169 | 4.82E-12 | 0.3308 | 651 295 | No |
|  | rs6573308 | T | C | 0.0248 | 0.004136 | 2.02E-09 | 0.3941 | 651 295 | No |
|  | rs7143933 | T | G | 0.0246 | 0.004045 | 1.19E-09 | 0.2615 | 651 295 | No |
|  | rs2978382 | T | C | 0.0232 | 0.003814 | 1.18E-09 | 0.5852 | 651 295 | No |
|  | rs4903203 | A | G | 0.0247 | 0.003688 | 2.13E-11 | 0.3237 | 651 295 | No |
|  | rs12436039 | T | C | 0.0332 | 0.005718 | 6.40E-09 | 0.8801 | 651 295 | No |
|  | rs4550384 | T | G | 0.0237 | 0.003751 | 2.63E-10 | 0.758 | 651 295 | No |
|  | rs710284 | T | C | 0.022 | 0.00373 | 3.67E-09 | 0.5797 | 651 295 | No |
|  | rs11845599 | A | G | -0.0274 | 0.003621 | 3.83E-14 | 0.6353 | 651 295 | No |
|  | rs59986227 | C | G | -0.0307 | 0.004334 | 1.40E-12 | 0.7493 | 651 295 | No |
|  | rs12442008 | T | C | 0.0285 | 0.00414 | 5.83E-12 | 0.2593 | 651 295 | No |
|  | rs4775086 | A | G | -0.0267 | 0.004472 | 2.37E-09 | 0.2363 | 651 295 | No |
|  | rs12442674 | A | C | 0.0229 | 0.003788 | 1.49E-09 | 0.7296 | 651 295 | No |
|  | rs1873958 | A | G | 0.0279 | 0.003361 | 1.04E-16 | 0.4088 | 651 295 | Yes |
|  | rs72773411 | A | G | 0.0293 | 0.005032 | 5.78E-09 | 0.1529 | 651 295 | No |
|  | rs12445235 | C | G | -0.0211 | 0.003546 | 2.69E-09 | 0.4098 | 651 295 | No |
|  | rs2304467 | C | G | -0.0237 | 0.003901 | 1.24E-09 | 0.6066 | 651 295 | No |
|  | rs11641239 | T | C | 0.0229 | 0.003729 | 8.20E-10 | 0.2854 | 651 295 | No |
|  | rs7203707 | A | C | -0.0197 | 0.003022 | 7.12E-11 | 0.5193 | 651 295 | No |
|  | rs4785296 | C | G | 0.0264 | 0.004157 | 2.14E-10 | 0.231 | 651 295 | No |
|  | rs3743794 | A | G | -0.0215 | 0.003534 | 1.18E-09 | 0.6073 | 651 295 | No |
|  | rs12927162 | A | G | 0.0561 | 0.004154 | 1.48E-41 | 0.7276 | 651 295 | Yes |
|  | rs1421085 | T | C | -0.0419 | 0.003392 | 4.65E-35 | 0.5935 | 651 295 | Yes |
|  | rs2550298 | T | C | -0.04 | 0.003621 | 2.28E-28 | 0.3811 | 651 295 | Yes |
|  | rs8044054 | T | C | 0.0307 | 0.003509 | 2.18E-18 | 0.3878 | 651 295 | No |
|  | rs72790386 | T | G | 0.0604 | 0.010279 | 4.20E-09 | 0.0338 | 651 295 | No |
|  | rs17604349 | A | G | -0.0374 | 0.003972 | 4.62E-21 | 0.1848 | 651 295 | Yes |
|  | rs1061032 | T | G | 0.0644 | 0.006151 | 1.19E-25 | 0.094 | 651 295 | Yes |
|  | rs11545787 | A | G | -0.0498 | 0.004168 | 6.66E-33 | 0.2482 | 651 295 | Yes |
|  | rs12950382 | A | G | 0.0234 | 0.004087 | 1.03E-08 | 0.7198 | 651 295 | No |
|  | rs4365329 | A | T | -0.0194 | 0.003328 | 5.59E-09 | 0.5383 | 651 295 | No |
|  | rs2011528 | T | C | -0.0329 | 0.004728 | 3.45E-12 | 0.8254 | 651 295 | No |
|  | rs3760381 | A | G | 0.0274 | 0.004176 | 5.33E-11 | 0.2544 | 651 295 | No |
|  | rs7225002 | A | G | -0.0179 | 0.003124 | 1.00E-08 | 0.5916 | 651 295 | No |
|  | rs12600452 | A | G | 0.0255 | 0.004065 | 3.53E-10 | 0.2015 | 651 295 | No |
|  | rs12051 | A | G | -0.0264 | 0.003484 | 3.54E-14 | 0.6119 | 651 295 | No |
|  | rs55846845 | A | G | -0.021 | 0.003045 | 5.34E-12 | 0.5177 | 651 295 | No |
|  | rs72829706 | A | G | 0.0563 | 0.008348 | 1.54E-11 | 0.96 | 651 295 | No |
|  | rs8072058 | A | T | -0.0281 | 0.004658 | 1.61E-09 | 0.7812 | 651 295 | No |
|  | rs412000 | C | G | -0.0224 | 0.003546 | 2.67E-10 | 0.555 | 651 295 | No |
|  | rs58681483 | A | G | 0.0352 | 0.005855 | 1.83E-09 | 0.918 | 651 295 | No |
|  | rs72841368 | A | T | -0.0301 | 0.004301 | 2.60E-12 | 0.8104 | 651 295 | No |
|  | rs2916148 | A | G | 0.0279 | 0.003505 | 1.71E-15 | 0.4522 | 651 295 | No |
|  | rs2580160 | A | G | 0.0283 | 0.004086 | 4.32E-12 | 0.5554 | 651 295 | No |
|  | rs62082402 | T | G | 0.05 | 0.005375 | 1.36E-20 | 0.1936 | 651 295 | No |
|  | rs1788784 | A | G | -0.0273 | 0.004157 | 5.15E-11 | 0.3442 | 651 295 | Yes |
|  | rs1013987 | T | C | -0.0291 | 0.003851 | 4.11E-14 | 0.4033 | 651 295 | No |
|  | rs4419127 | A | G | 0.0444 | 0.003735 | 1.35E-32 | 0.6622 | 651 295 | Yes |
|  | rs9950528 | A | G | -0.0235 | 0.003819 | 7.57E-10 | 0.6505 | 651 295 | No |
|  | rs12969848 | T | C | 0.0355 | 0.003539 | 1.12E-23 | 0.5313 | 651 295 | Yes |
|  | rs9956387 | A | T | -0.0198 | 0.003397 | 5.57E-09 | 0.4953 | 651 295 | No |
|  | rs4800998 | A | T | 0.0394 | 0.004984 | 2.69E-15 | 0.1827 | 651 295 | Yes |
|  | rs9964420 | A | C | -0.0488 | 0.003645 | 6.97E-41 | 0.2983 | 651 295 | Yes |
|  | rs11152350 | A | C | -0.0281 | 0.003615 | 7.63E-15 | 0.467 | 651 295 | No |
|  | rs34329963 | T | C | -0.0318 | 0.005328 | 2.39E-09 | 0.113 | 651 295 | No |
|  | rs1025601 | T | C | -0.0218 | 0.00374 | 5.57E-09 | 0.3855 | 651 295 | No |
|  | rs10402849 | T | C | 0.0259 | 0.004165 | 5.04E-10 | 0.2002 | 651 295 | No |
|  | rs36055559 | A | G | -0.036 | 0.00501 | 6.69E-13 | 0.166 | 651 295 | No |
|  | rs7248205 | T | C | 0.0267 | 0.003572 | 7.78E-14 | 0.6003 | 651 295 | No |
|  | rs9636202 | A | G | -0.026 | 0.003749 | 4.06E-12 | 0.2662 | 651 295 | No |
|  | rs73026775 | A | G | -0.0335 | 0.005709 | 4.41E-09 | 0.1246 | 651 295 | No |
|  | rs4804951 | A | G | 0.0234 | 0.003806 | 7.83E-10 | 0.3306 | 651 295 | No |
|  | rs56113850 | T | C | -0.0229 | 0.003449 | 3.15E-11 | 0.4231 | 651 295 | No |
|  | rs58876439 | A | G | 0.0474 | 0.006806 | 3.31E-12 | 0.0674 | 651 295 | No |
|  | rs11670534 | T | C | -0.0305 | 0.004689 | 7.83E-11 | 0.1614 | 651 295 | No |
|  | rs6131805 | T | G | 0.0257 | 0.003643 | 1.72E-12 | 0.4022 | 651 295 | No |
|  | rs6131942 | A | G | -0.0263 | 0.003112 | 2.89E-17 | 0.418 | 651 295 | Yes |
|  | rs1474754 | A | G | -0.0214 | 0.003623 | 3.51E-09 | 0.2636 | 651 295 | No |
|  | rs6047481 | A | T | 0.0253 | 0.004084 | 5.81E-10 | 0.6709 | 651 295 | No |
|  | rs1737893 | T | C | -0.0253 | 0.003728 | 1.15E-11 | 0.3805 | 651 295 | No |
|  | rs2072727 | T | C | 0.0282 | 0.003427 | 1.89E-16 | 0.4327 | 651 295 | Yes |
|  | rs57236847 | C | G | 0.0269 | 0.004373 | 7.69E-10 | 0.6029 | 651 295 | No |
|  | rs695459 | T | C | -0.022 | 0.00368 | 2.25E-09 | 0.3906 | 651 295 | No |
|  | rs28459838 | T | C | 0.0266 | 0.004183 | 2.03E-10 | 0.2369 | 651 295 | No |
|  | rs118047999 | C | G | 0.0236 | 0.003982 | 3.10E-09 | 0.2459 | 651 295 | No |
|  | rs139911 | T | C | -0.0336 | 0.003644 | 2.94E-20 | 0.5722 | 651 295 | No |
|  | rs9611597 | A | T | 0.0374 | 0.005098 | 2.20E-13 | 0.8371 | 651 295 | No |
|  | rs6007594 | A | G | -0.025 | 0.003859 | 9.26E-11 | 0.2644 | 651 295 | No |
